# Adults With Complex Congenital Heart Disease: Cerebrovascular Considerations for the Neurologist

**DOI:** 10.3389/fneur.2019.00329

**Published:** 2019-04-04

**Authors:** Jonathan M. C. Smith, Jason G. Andrade, Derek Human, Thalia S. Field

**Affiliations:** ^1^M.D. Senior Pediatric Neurology Resident, University of British Columbia, Vancouver, BC, Canada; ^2^FRCPC Clinical Associate Professor of Cardiology, University of British Columbia, Vancouver, BC, Canada; ^3^FRCPC, Clinical Professor of Pediatric Cardiology, BC Children's Hospital, University of British Columbia, Vancouver, BC, Canada

**Keywords:** Congenital Heart Disease (CHD), Adults with Congenital Heart Disease (ACHD), Cerebrovascular disease, Cardiovascular Disease, stroke, cognition, cyanotic, fontan

## Abstract

As infant and childhood mortality has decreased in congenital heart disease, this population is increasingly reaching adulthood. Adults with congenital heart disease (ACHD) represent a group with increased risk of stroke, silent brain infarcts, and vascular cognitive impairment. Cyanotic and other complex cardiac lesions confer the greatest risk of these cerebrovascular insults. ACHD patients, in addition to having an increased risk of stroke from structural cardiac issues and associated physiological changes, may have an accelerated burden of conventional vascular risk factors, including hypertension and impaired glucose metabolism. Adult neurologists should be aware of the risks of clinically evident and subclinical cerebrovascular disease in this population. We review the existing evidence on primary and secondary stroke prevention in individuals with complex congenital heart disease, and identify knowledge gaps in need of further research, including treatment of acute stroke in this population. Multisystemic genetic syndromes are outside the scope of this review.

## Introduction

Congenital Heart Disease (CHD) encompasses a wide range of developmental cardiac lesions, including simple left-to-right shunts, obstructive lesions, and mixing disorders causing cyanosis. Incidence is approximately 8/1,000 live births in high-income countries (1). Infant and childhood mortality from CHD was 80% in the 1950s. However, owing to improvements in diagnostics, medical therapy, and surgical techniques, over 90% of children born with CHD now reach adulthood (2–4). Adults with congenital heart disease (ACHD) represent a group at increased risk of stroke, silent brain infarcts, and vascular cognitive impairment (5–9). As their longevity improves, ACHD are an increasingly relevant population for adult neurologists (3). In this review, we focus on complex congenital heart disease in individuals without multisystemic genetic syndromes.

## Epidemiology and Classification of CHD and Changing Demographic Profiles

Congenital heart lesions are classified anatomically (simple and complex) and physiologically (cyanotic and acyanotic) (10) (Table 1). However, anatomical and physiological severity are not always correlated. Thus, in contrast to other disease states, the ACHD Anatomic and Physiological classification considers the shifting and complex interplay between these factors (native anatomy, state of surgical repair [unrepaired, repaired, or palliated], and current physiology) to guide prognosis, management, and resource utilization.

**Table 1:** Classification of congenital heart lesions (10).

**Simple**	**Native disease** Isolated small atrial septal defect (ASD) Isolated small ventricular septal defect (VSD) Mild isolated pulmonic stenosis	**Repaired conditions** Previously ligated or occluded ductus arteriosus Repaired secundum ASD or sinus venosus defect without significant residual shunt or chamber enlargement Repaired VSD without significant residual shunt or chamber enlargement
Moderate Complexity	**Repaired or unrepaired conditions** Anomalous pulmonary venous connection, partial or total Atrioventricular (AV) septal defect (partial or complete) Coarctation of the aorta Ebstein anomaly (including mild, moderate, and severe variations) Infundibular RV outflow obstruction Repaired tetralogy of fallot (TOF) VSD with associated abnormality and/or moderate or greater shunt	**Valve (or near-valve) conditions** Pulmonary valve regurgitation (moderate or greater) Pulmonary valve stenosis (moderate or greater) Subvalvular aortic stenosis (excluding hypertrophic cardiomyopathy) Supravalvular aortic stenosis Congenital aortic valve disease Congenital mitral valve disease
Great Complexity (or Complex)	Cyanotic congenital heart defect (unrepaired or palliated, all forms) Double-outlet ventricle Fontan procedure Interrupted aortic arch Truncus arteriosus	Single Ventricle (including double inlet left ventricle, tricuspid atresia, hypoplastic left heart) Pulmonary atresia Mitral atresia Transposition of the great arteries (TGA)

In adulthood, the most frequently encountered CHD lesion is a simple left-to-right shunt (11). Most commonly these are Atrial Septal Defects (ASD, 10% of CHD but up to 40% of CHD presenting in adulthood) and Ventricular Septal Defects (VSD, <5% of CHD in the US but 1/3 of CHD presenting in childhood) (12, 13). A recent review on stroke in young adults discusses patent foramen ovale (PFO)-associated stroke (14). Here we focus primarily on cyanotic and more complex lesions, which are associated with a greater risk of cerebrovascular disease (5–8, 15).

About one-third of children with CHD will have a multisystemic genetic syndrome (16). This figure drops to 13% in the ACHD population (17). Common genetic syndromes associated with ACHD include Trisomy 21, Marfan syndrome, 22q11 microdeletion, Turner syndrome and Williams syndrome (16, 17). ACHD as part of a multisystemic genetic syndrome is independently associated with poorer neurodevelopmental outcomes (9, 16) and greater cardiac lesion severity (17). This group constitutes a complex and heterogeneous population outside of the scope of this review. In addition to well-defined genetic syndromes, recently described microdeletions and epigenetic modifiers may also affect a large proportion of individuals with CHD (16, 18, 19).

**Figure 1:**
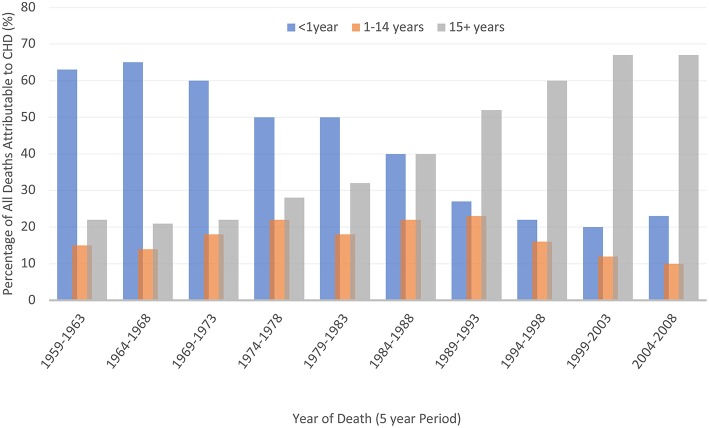
Improved survival of people affected by CHD [data from Knowles et al. (23)].

Prevalence of CHD has increased from 0·6/1,000 live births in the 1930s to 9·1/1,000 after 1995 (1). There are now over 1·4 million American adults with CHD, representing over two thirds of the total CHD population (3). Prevalence of ACHD is 6·16/1,000, and 0·68/1,000 for severe lesions (5–8, 20, 21). This represents a 63% increase over the last two decades (3), with further increases estimated at 1-5%/year (2, 22) (Figure 1).

## Anatomy and Physiology of Surgically Repaired Complex Congenital Heart Lesions

**Figure 2:**
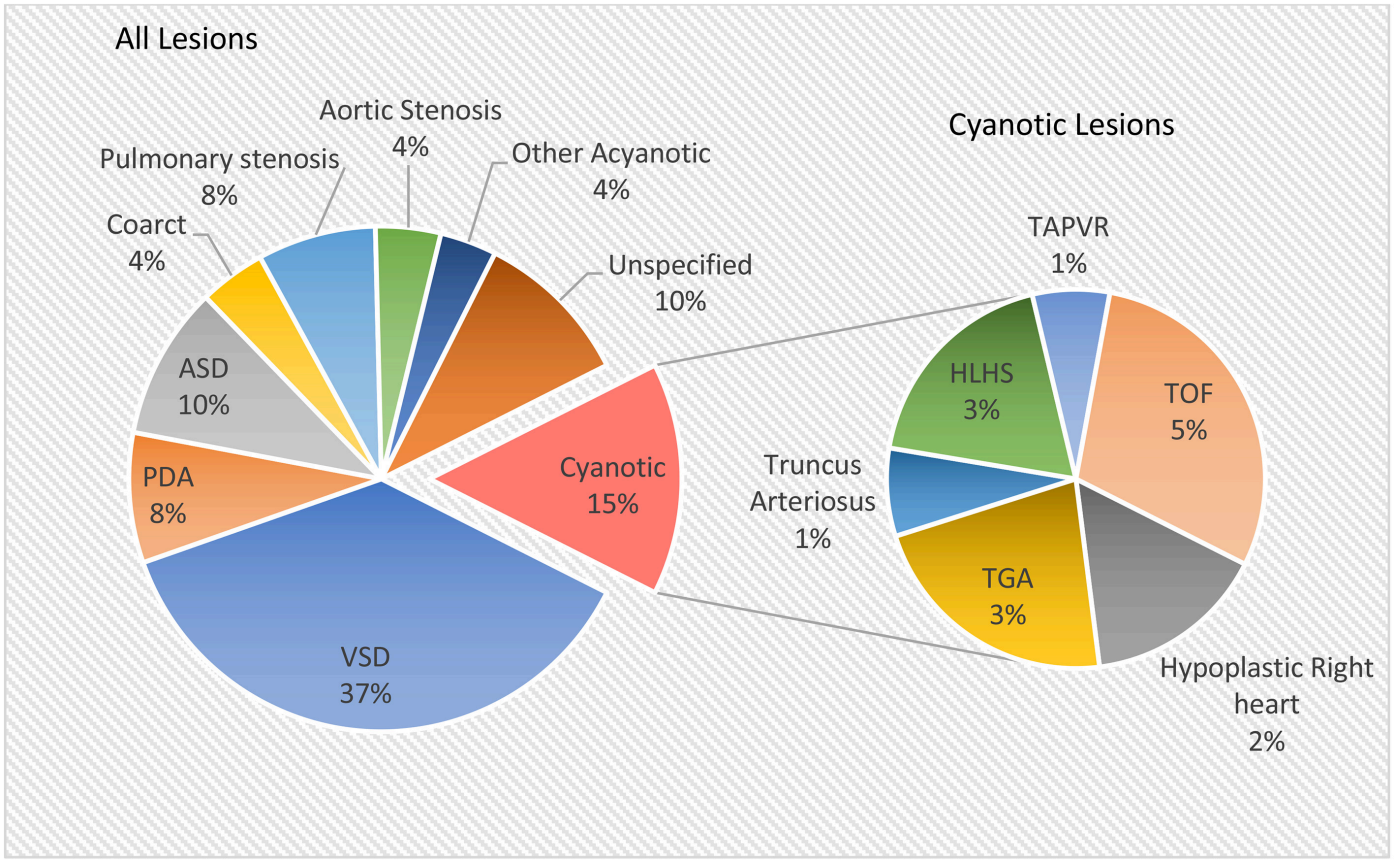
Relative prevalence of congenital heart lesions [data from Hoffman and Kaplan (13)].

While some forms of simple CHD may be discovered in adulthood (e.g., ASD), most individuals with complex CHD will have undergone surgical correction in early life (12, 24). The most common complex post-operative conditions seen in adulthood are Tetralogy of Fallot, combined atrioventricular septal defect, and transposition of the great arteries (11, 24). Prevalence is increasing for complex single ventricle lesions (e.g., hypoplastic left and right heart syndromes) as more individuals with Fontan repairs reach adulthood (25). Complex saturated-desaturated blood mixing disorders include total anomalous pulmonary venous return, and truncus arteriosus (Figure 2). These are less common, and those affected may have multiple surgical procedures in childhood (11, 26–28).

In some simple left-to-right shunt lesions like ASD and VSD, greater-than-normal pulmonary blood flow may, over time, result in pulmonary hypertension. If pulmonary vascular resistance exceeds systemic vascular resistance, the shunt direction can reverse, resulting in cyanosis; this is called Eisenmenger syndrome (12).

While some patients may undergo full surgical correction, others will require palliative procedures. A palliative procedure does not correct abnormal anatomy and function, but aims to minimize cyanosis and heart failure (29). Some patients may have corrective surgery following a period of palliation (12, 29), and others with very complex lesions may only be suitable for long-term palliative circulations (29, 30).

### Physiological Changes After Fontan Repairs

A Fontan circulation is the final common pathway used in the palliative management of single ventricle physiology (e.g., univentricular congenital heart lesions, tricuspid atresia, and double inlet left ventricle) (30). Broadly, it has been developed with four variations on anastomotic sites (30) (Table 2). The single-ventricle circulation may be a morphologically right or left ventricle, ejecting to the systemic circulation while maintaining diastolic function to promote passive pulmonary blood flow.

**Table 2:** Four types of Fontan circulations and their relevant anatomy (30).

**Type of Fontan**	**Connections**	**Anatomy**
Atriopulmonary (Fontan and Baudet, Kreutzer et al. 1960s-70s)	Right atrial appendage connected to PA	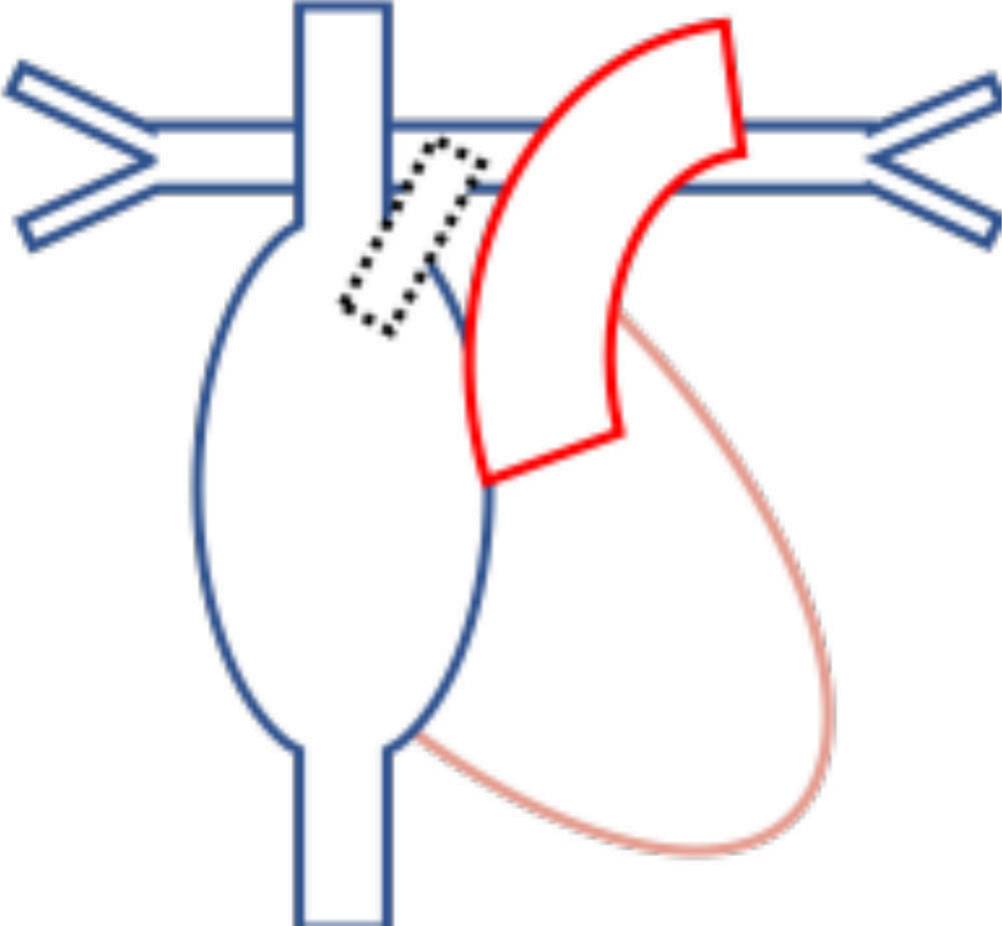
Atrioventricular (Bjork et al. late 1970s	Right atrial appendage to at least moderately sized residual right ventricle	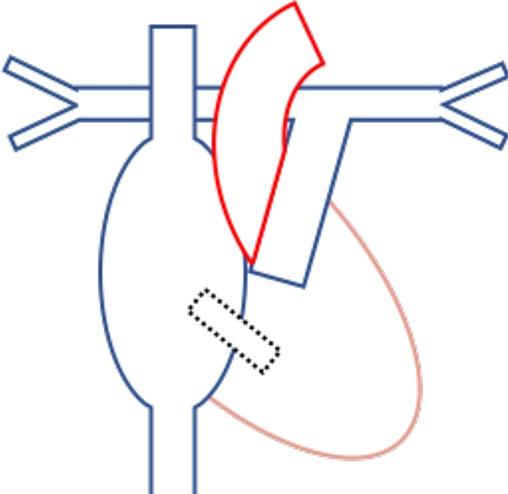
Lateral Tunnel (De Leval et al. late 1980s)	SVC to PA, IVC to PA via intracardiac tunnel using lateral wall of right atrium as part of conduit	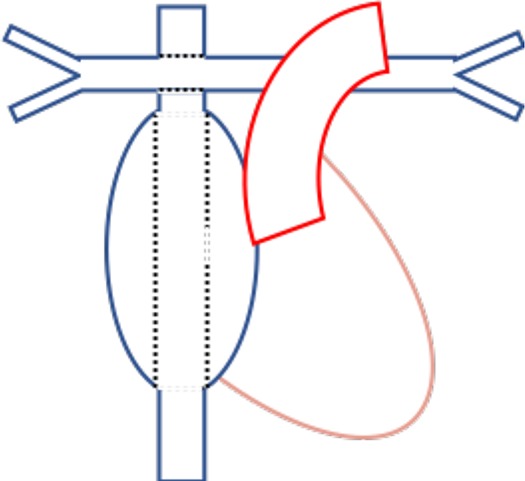
Extracardiac (Marcelletti et al. 1990s)	SVC to PA, IVC to PA via extracardiac conduit	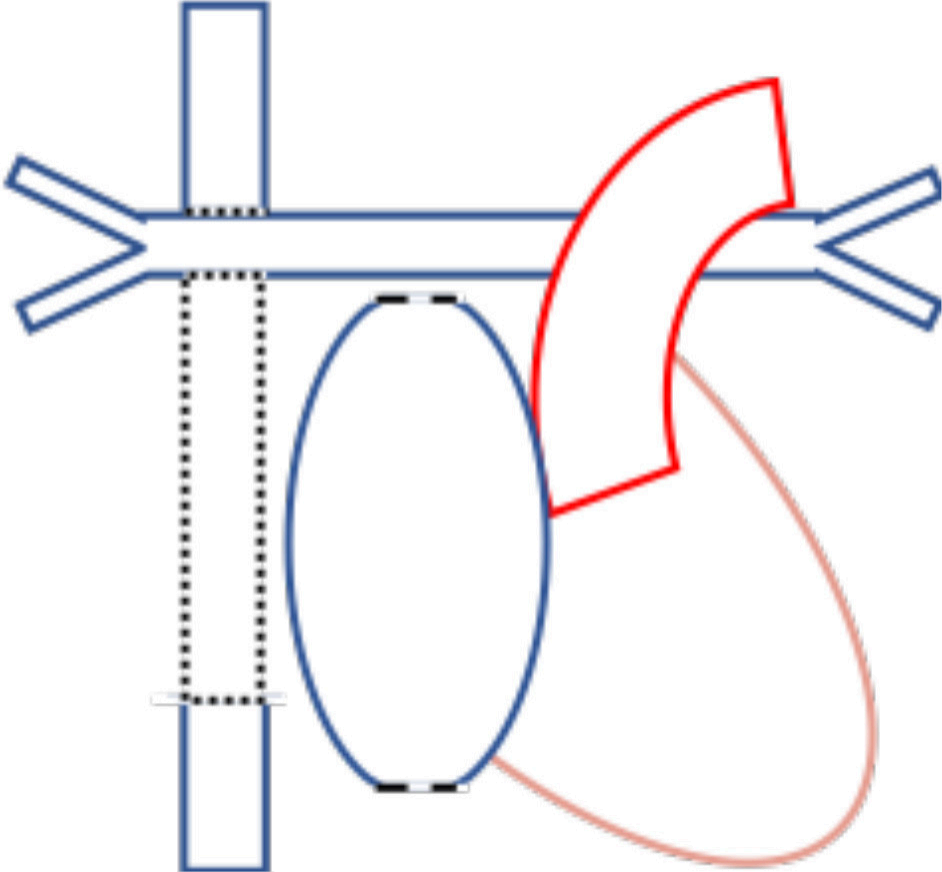

*PA, pulmonary artery; SVC, superior vena cava; IVC, inferior vena cava*.

The final Fontan physiology is complex, with long-term consequences related to the obligatory elevation in central venous pressure and reduced cardiac output (30). These morphologic and physiologic features contribute to stroke risk through multiple mechanisms. The abnormal ventricular morphology contributes to ventricular dysfunction. Prolonged exposure to elevated intracardiac pressures leads to atrial dilatation and fibrosis, with subsequent development of cardiac dysrhythmia (30). Further, the low, fixed, non-pulsatile cardiac output in Fontan circulations may contribute to impaired nitric oxide release and subsequent endothelial pathway dysfunction, increasing both systemic and pulmonary vascular resistance (30). Portal hypertension may cause ascites, cirrhosis, and protein-losing enteropathy, contributing to coagulopathy (30).

## Stroke in ACHD

### Ischemic Stroke

CHD, particularly cyanotic lesions, confers an increased risk of stroke and silent brain infarcts in adults (5–7) (Table 3). A prospective Swedish registry study found an almost 11-fold increased risk of ischemic stroke in individuals with CHD (excluding PFO identified age <underlin> e>18) compared to age-matched controls (end follow-up median age 29) (6). This risk was highest in patients with the most complex lesions (Table 3). Those with CHD were at a greater risk of ischemic stroke in the presence of comorbid hypertension (HR 3·89; 95%CI 2·44–6·22), heart failure (6·94; 95%CI 4·96–10·34), and atrial fibrillation (AF) (2·94; 95%CI 1·78–4·83), but not diabetes, all of which were more prevalent in the CHD cohort than controls at any time point.

**Table 3:** Ischemic stroke in ACHD.

**Study**	**Population**						
Mandalenakis et al. (6)	25,000 ACHD patients (median age 28.6), Swedish database data.	Lesion type	All CHD		High complexity lesions	Atrial Level Shunt (ASD or PFO)	Aortic coarctation	Double Inlet Ventricle
		Risk of Ischemic Stroke–HR vs. age-matched healthy control (95% CI)	10.76 (8.49–13.63)		12.22 (7.93–18.85)	10.00 (2.02–49.55)	12.86 (4.79–34.56)	4.49 (1.56–12.93)
Lanz et al. (5)	29,638 ACHD patients aged 18–64, Quebec province-wide admin data	Age (years)	18–24		25–34	35–44	45–54	55–64
		Ischemic Stroke Rate per 1,00,000 patient years (95% CI) - Men	16 (5–38)		63 (36–102)	199 (141–274)	287 (213–377)	304 (227–400)
		Ischemic Stroke Rate per 1,00,000 patient years (95% CI) - Women	29 (15–52)		43 (25–67)	97 (65–139)	196 (140–265)	292 (217–384)
Hoffman et al. (7)	23,153 ACHD patients aged 16–91 (mean 36.4), Canadian and European ACHD databases	Lesion Type	TOF	TGA	Open ASD	Fontan circulation	Eisenmenger cyanotic lesion	Non-Eisenmenger cyanotic lesion
		Prevalence Estimates of Ischemic Stroke	2.4%	3.2%	4.0%	4.1%	5.1%	23.3%

A retrospective study in over 29,000 ACHD patients aged 18–64 found a cumulative risk of ischemic stroke of 6·1% in women and 7·7% in men (5). Half (47%) had septal defects (excluding PFO) or patent ductus arteriosus; 17% severe lesions. When stratified by cardiac lesion, the highest risks for stroke in adulthood were in those with complex (8·9%; 95%CI 6·0–11·5%) and left-sided (9·5%; 95%CI 7·8–11·1%) lesions (Table 3). In this cohort, comorbid diabetes (OR 2·33; 95%CI 1·66–3·28), heart failure (OR 5·94; 95%CI 3·49–10·14), and recent myocardial infarction (OR 8·38; 95%CI 1·77–39·58) were associated with a risk of ischemic stroke. Atrial arrhythmias, dyslipidemia, and hypertension were not.

A retrospective analysis including 23,000 patients derived from the two studies described above further assessed the association between complex lesion type and ischemic stroke (7). Over a mean follow-up of 36·3 years (842,769 patient-years), overall prevalence of stroke in the CHD group was 2·0%. Rates differed by cardiac lesion, ranging from 10 to 100 times that of age-matched controls. Non-Eisenmenger cyanotic lesions in particular were at extremely high risk (23%).

Variability with regards to identified risk factors between studies may relate in part to studies' designs and their associated limitations, including retrospective design in some cases, use of administrative data, and a focus on clinically evident events.

#### Ischemic Stroke and White Matter Hyperintensities on Neuroimaging

The risk of cerebral infarction is likely underestimated as most studies have focused on clinically evident events (31–33). In one neuroimaging cohort of 156 adolescents with Fontan circulations, 13% had infarcts on neuroimaging, but only 60% of those had a clinical history of stroke (34). Whether childhood infarct burden predicts stroke recurrence in adulthood is not known. The burden of ischemic brain disease in CHD may begin in very early life. In neonatal patients with cyanotic lesions, longer wait-times for palliative procedures are associated with increased incidence of white-matter injury (35). This risk may further accumulate with increasing numbers of cardiac surgeries and catheterizations, particularly in those who undergo re-operation (33).

A cross-sectional neuroimaging evaluation of 72 clinically stable ACHD adults (mean age 40 ± 14) with persistent cyanotic cardiac anatomy (70% Eisenmenger, 18% single ventricle physiology) found that 47% had evidence of prior ischemic stroke and 65% had white matter hyperintensities on MRI (8). Of those, most (42%) had isolated lacunar infarcts, 29% isolated cortical infarcts, and both in 29%. In those with infarcts, 53% had multiple ischemic lesions. Only 21% of those with infarcts had a clinical history of stroke.

### Hemorrhagic Stroke

Hemorrhagic stroke risk in ACHD is not as well characterized as that of ischemic stroke, and the effect of antithrombotic use on hemorrhage risk in this population is not known. A large administrative data study in 30,000 ACHD patients aged 18–64 found a cumulative risk of hemorrhagic stroke of 0.8% (95%CI 0·4–1·2) in women, and 1·3% (95%CI 0·8–1·8) in men (5). There was no direct comparison to rates in healthy controls, nor an evaluation of predictors of hemorrhagic stroke.

A registry study of 22,000 ACHD patients matched ten to one with random controls from the general population found an increased rate of intracerebral hemorrhage (ICH) as well as subarachnoid hemorrhage (SAH) in the ACHD group over 27 years of follow-up (36). For ACHD, incidence rate ratios (IRR) for intracerebral (ICH) and subarachnoid hemorrhage (SAH) were 8·23 (95%CI 6·0–11·2) and 7·64 (95%CI 5·41–10·7), respectively. The greatest risk of ICH was observed in patients with complex lesions (IRR 16·5; 95%CI 5·63–51·2). Aortic coarctation had the highest risk of SAH (IRR 17·3; 95%CI 6·33–51·8), possibly due to the combination of premature hypertension and congenital vascular abnormalities leading to an increased incidence (approximately 10%) and earlier onset of berry aneurysms (37, 38). Unfortunately, the role of screening for aneurysms in adults with aortic coarctation is not well characterized, and it is unclear as to whether typical risk stratification approaches based on aneurysm size and location apply in this population (39).

### Stroke Mechanisms and Risk Factors in ACHD (Figure 3)

Adult CHD patients, in addition to having an increased risk of stroke related to the underlying structural cardiac issues (and associated dysrhythmias and coagulation abnormalities), may also have an accelerated burden of conventional vascular risk factors such as hypertension and impaired glucose metabolism (9, 20). The impact of these risk factors is increasingly significant as the lifespan of patients with ACHD continues to improve.

**Figure 3:**
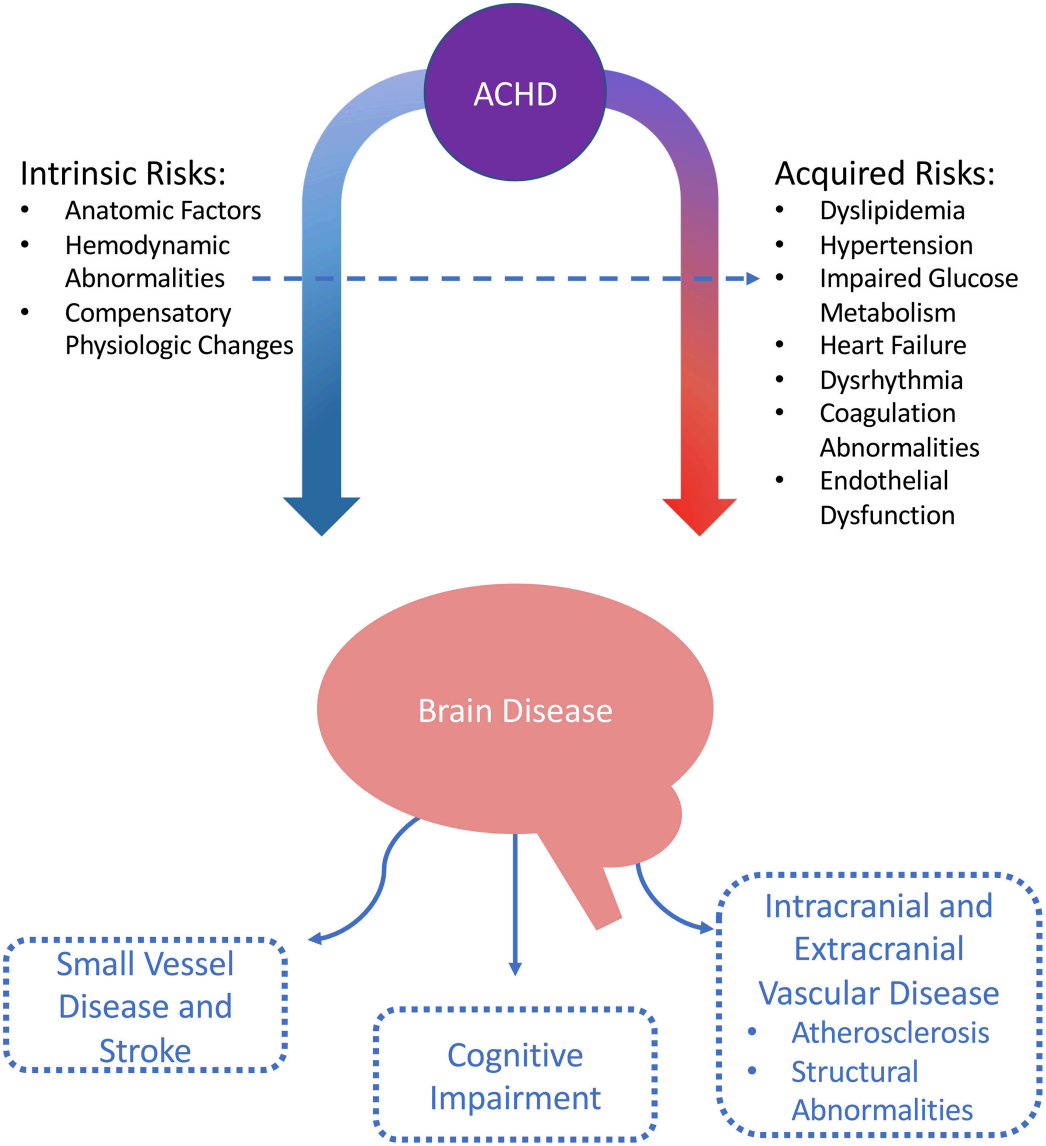
Mechanisms of cerebrovascular disease in ACHD.

#### Chamber Dilatation

In many CHD lesion types, cardiac chambers are exposed to abnormal flow patterns and atypical pressure gradients. This is most often as a result of the chambers being exposed to pressures, or performing functions, which are otherwise atypical (12, 20, 30). For example, in Hypoplastic Left Heart Syndrome, the patient will have an anatomic right ventricle (which is usually a high volume-low pressure chamber) pumping against systemic vascular resistance.

These abnormal hemodynamic flow patterns and pressure gradients are present in the pre- and post-operative state, and can be extreme in palliated circulations (30). Pressure- and volume-loading of the cardiac chambers results in chamber dilatation and impaired mechanical function, leading to stasis and increased potential for thrombus formation (20). Pressure and volume stress can cause replacement fibrosis, with subsequent distortion of electrophysiologic chamber properties leading to dysrhythmias and conduction block (20, 40).

#### Dysrhythmias

A significant proportion of ACHD experience dysrhythmias throughout their lifetime, and prevalence increases with age (41). Arrhythmias are the most frequent reason for hospitalization in ACHD and are a leading cause of death. In general, presence of an atrial tachyarrhythmia in ACHD confers a greater risk of thromboembolism than in the general population (42).

Arrhythmia types encountered in CHD are broad, with all mechanisms of dysrhythmia represented. A significant proportion are paroxysmal (40). The arrhythmias may reflect primary myocardial disease related to native CHD pathology, a malformed or disrupted native conduction system (e.g., sinus or atrioventricular node), abnormal hemodynamic flow patterns and pressure gradients, or postoperative sequelae (20, 40, 41).

A population-based analysis of over 38,000 ACHD found that after survival to 18 years, cumulative risk of developing an atrial arrhythmia by age 75 was 47%. This risk increased to 63% with complex CHD lesions (43). Intra-atrial reentry tachycardia (IART or “flutter”) and atrial fibrillation (AF), two of the most commonly encountered tachyarrhythmias in ACHD, were associated with a significant risk of stroke or heart failure (HR 2·21; 95%CI 2·07–2·36) as well as mortality (HR 1·47; 95%CI 1·37–1·58) (43).

#### Paradoxical Emboli

Most shunt lesions have left-to-right flow, though shunt reversal may occur temporarily in the context of exercise or more permanently in the presence of pulmonary arterial hypertension (e.g., shunt reversal in Eisenmenger syndrome). Patients with cyanotic anatomy necessarily have a right-to-left shunt. While not all shunts represent potential conduits for venous thromboembolism, many found in cyanotic cardiac lesions have large connections between the pulmonic and systemic circulations that potentiate risk of paradoxical embolism (20). This risk is increased in the setting of atrial tachyarrhythmias such as atrial fibrillation, or in the presence of foreign material such as a pacemaker (20, 44).

#### Hyperviscosity and Hypercoagulability

Patients with significant sustained hypoxemia develop compensatory erythrocytosis, which can increase thrombotic risk from hyperviscosity (20, 45). The elevation in hematocrit causes increased whole blood viscosity which imposes shear stresses on vessel walls (46). Concomitant iron deficiency worsens the oxygen carrying capacity of red blood cells, and reduces cell deformability, causing intravascular sludging and further increased risk of vessel occlusion (20, 45).

There is also an association between CHD and thrombophilias (both genetic and acquired). One case series identified a “prothrombotic state” in 36% of 135 pediatric patients with CHD and history of stroke (47). A second case series identified congenital thrombophilia in seven of 21 pediatric CHD patients with a history of stroke, most commonly antithrombin deficiency (31). The methylenetetrahydrofolate reductase (MTHFR) gene mutation, associated with venous thromboembolism, is also associated with CHD (48, 49). The mechanisms and directionality of the relationship between CHD and genetic thrombophilia is not well understood.

ACHD may have evidence of both thrombophilia and coagulopathy, demonstrating increased platelet aggregation, decreased levels of antithrombin III, thrombomodulin, alpha 2 antiplasmin, protein C and S activities, and significantly increased thrombin-antithrombin complex, and alpha-2 antiplasmin inhibitor complex (50, 51). Adults with cyanotic lesions are at increased risk of bleeding and have been shown to have dysfunctional clot formation and breakdown (52).

### Conventional Vascular Risk Factors

Although rates of some conventional vascular risk factors, including hypertension and diabetes, are increased in CHD as compared to the general population, individuals with persistent cyanotic lesions may have lower rates of some atherosclerotic risk factors. Possible mechanisms for this difference are postulated to be secondary to biochemical alterations from chronic hypoxemia, including secondary erythrocytosis, thrombocytopenia, and increased nitric oxide (53). Still, vascular risk persists in this group secondary to additional risks, such as endothelial dysfunction and abnormal vascular microstructure (46, 53–58). Existing neuroimaging evidence suggests that prevalence of incidental brain infarcts and white matter hyperintensities are high with cyanotic CHD (8).

#### Hypertension

Irrespective of the underlying pathology, the rates of hypertension are increased in ACHD as compared with healthy controls (59). In a Swedish registry study of over 25,000 patients, both children and young adults with CHD (median age 29) had a greater prevalence of hypertension than controls (7·1 vs. 0·5%), and were at a 3-fold greater risk of ischemic stroke in the presence of hypertension (HR 3·89; 95%CI 2·44–6·22) (6). In another large database study in CHD (median age 50), subjects with a history of stroke were more likely to be hypertensive compared with controls (31·2 vs. 22·2%, HR 1·73; 95%CI 1·32–2·25) (5).

Aortic coarctation is associated with a particularly high risk of hypertension. A systematic review identified a median prevalence of hypertension of 32·5% (range 25–68%) at long-term follow-up after coarctation repair (45). Prevalence may be as high as 90% in patients over the age of 50 (60, 61). In addition to the primary pathology (e.g., anatomical vascular resistance in uncorrected patients or post-surgical patients with re-coarctation), postulated mechanisms for hypertension in the repaired coarctation population relate to chronic structural vascular changes, reduced baroreceptor sensitivity, dysfunction of the renin-angiotensin system, and endothelial dysfunction (62).

Conversely, cyanotic patients have been found to have depressed diastolic and systolic blood pressures. One small study comparing 54 patients with cyanotic lesions (mean age 38) to age-matched controls found significantly lower systolic and blood pressures (113 ± 14 mmHg vs. 124 ± 12 mmHg; 71 ± 9 mmHg vs. 76 ± 9 mmHg, respectively) in the ACHD group (63).

#### Dyslipidemia

There is no evidence to suggest that rates of dyslipidemia are higher in ACHD as compared to the general population (64, 65). One UK single-center study of 250 ACHD patients (mean age 51) found the prevalence of dyslipidemia to be 19·1%, with dyslipidemia being a strong risk factor for coronary artery disease in this cohort (OR 9·08; 95%CI 3·56–24·54) (66). Prevalence of dyslipidemia, however, decreased with increasing cardiac lesion complexity. Data from the Quebec Congenital Heart Disease Database found a prevalence of dyslipidemia in 27% of patients over the age of 65, which is lower than estimates for the general population (57, 67).

Cyanotic patients have a lower total cholesterol than acyanotic patients (53, 68, 69). This effect persists even in patients who have been surgically corrected to an acyanotic circulation (58).

#### Impaired Glucose Metabolism and Diabetes

Population-based cohort studies suggest increased rates of diabetes and impaired glucose metabolism in ACHD as compared to healthy controls (57, 66, 68–70). The risk of metabolic syndrome also appears to be higher in the ACHD population (10, 22, 71). The prevalence in older adults >65 years of age with ACHD has been found to be as high as 16%. Risk of developing diabetes is also higher in ACHD over time (72). A population-based cohort study comparing 5,149 ACHD patients (aged 30 and above) with the general population found that ACHD were more likely to develop Type II DM by age 45 (HR 1·4; 95%CI 1·1–1·6), and cyanotic ACHD patients were likelier to develop Type II DM than acyanotic patients (HR 1·9; 95%CI 1·1–3·3) (73). The literature linking cyanotic CHD and diabetes, however, is not consistent. A cross-sectional study of 205 ACHD patients (mean age 24) vs. age-matched healthy controls found a significantly increased prevalence of DM in post-Fontan and other ACHD, but not in cyanotic patients (74).

There are both genetic and epigenetic, in addition to acquired, risk factors that may account for the association between CHD and diabetes. Maternal diabetes mellitus [both gestational and pre-gestational (75)] is associated with an increased risk of CHD in the fetus, with evidence of cardiac protein expression from certain genes being altered in animal models of maternal hyperglycemia (76). Increased prevalence of diabetes in CHD patients could in part represent a shared heritability for diabetes between mother and offspring with CHD (77). Additionally, risk may be acquired through increased susceptibility for metabolic syndrome through decreased physical activity (71). It has also been noted that chronic hypoxia from respiratory causes leads to decreased insulin secretion and sensitivity and thus posited that a shared mechanism could underlie the association between cyanotic heart disease and diabetes (73).

#### Lifestyle Factors

The increased vascular risks associated with CHD may be further affected by prevalence of lifestyle-related and psychosocial risk factors that are associated with increased risk of stroke in other populations (10). Exercise capacity has a significant impact on quality of life in people with ACHD (78). There is conflicting evidence regarding participation in physical exercise in ACHD cohorts, with some studies reporting lower levels of physical activity, though this is complicated by heterogenous exercise capacity associated with different cardiac lesions. There are reports that rates of metabolic syndrome in ACHD are higher than in age-matched controls (71). though prevalence of overweight and obese individuals appears to be comparable to that of the general population (79) and decreased participation in physical activity is proposed to underlie some of this disparity.

Rates of smoking and recreational drug use seem to be lower in the ACHD population than in the general population (53, 57, 78). Psychosocial factors associated with increased vascular risk in other populations, however, including anxiety, depression and lower educational attainment, (10, 78, 80) are more common in people with ACHD as compared to the general population.

## Cognitive Impairment in CHD and Proposed Mechanisms

As the life expectancy of patients with CHD continues to improve, the potential burden of cerebrovascular disease increases. Knowledge to date of neurodevelopmental sequelae in CHD and potential mechanisms for neurodegeneration over the lifespan have been reviewed recently (9). Even in the absence of an identified genetic syndrome, the presence of neurodevelopmental sequelae in a large proportion of children with CHD is well-documented (9, 16). The relationship between neurological and cardiac dysmaturation may be associated in some cases with common genetic (16, 81) or epigenetic (82, 83). alterations impacting gene expression of both heart and brain during fetal development, but there are also multiple other potential mitigating factors including disruptions to fetal brain oxygenation and perfusion from alterations to normal circulatory patterns (84–87), placental insufficiency (88), and increased risk of spontaneous preterm birth in CHD (89). Beginning *in utero*, the brains of fetuses with CHD can differ in structural, neurochemical, and metabolic makeup. There can be delays in structural maturation, structural white matter disorganization, decreased oxygen delivery, and an altered chemical and metabolic microenvironment (16, 34, 91).

Three quarters of children with moderate, and less than half of those with severe CHD are free of any neurodevelopmental impairment (90). While the incidence or prevalence of cognitive impairment in ACHD has been described in some studies, those with known neurodevelopmental issues are excluded from these cohorts. The actual prevalence of neurodevelopmental normalcy in the ACHD population is not known. While congenital brain abnormalities predispose individuals with CHD to further cognitive decline and dementia, even the proportion of CHD patients who are neurodevelopmentally normal face a greater lifetime risk and earlier onset of cognitive impairment and dementia due to the accumulation of endogenous and periprocedural vascular insults over time (9, 15, 91).

Overall, ACHD are significantly more likely to have impairment in social achievements. As compared to controls, ACHD under 40 years of age attain lower levels of education, are more likely to be unemployed, and are 20% less likely to be in a relationship (70).

There is a small body of literature regarding cognitive function in ACHD. A group of 48 adults with moderate and severe lesions (18–49 years of age) who had undergone cardiac surgery before 5 years of age underwent standardized cognitive testing (92). Patients with a known genetic syndrome were excluded. Patient with severe, but not moderate, lesions had significantly worse scores in multiple domains, including psychomotor speed, processing speed, complex attention, reaction time, and the overall neurocognitive index. The severe lesion group also had a significantly higher incidence of moderate neurocognitive impairment compared with norms.

In another cross-sectional study of psychologic and cognitive function in ACHD, 310 subjects (mean age 33·3 years) underwent neuropsychological assessment (93). Patients with a known genetic syndrome, history of stroke, or intellectual, learning or physical disability were excluded. Among subjects, 41% showed impaired performance on at least three tests of cognitive domains as compared to an expected 8% in healthy controls.

Differences in cognition are also seen in the aging ACHD population. A large hospital registry study evaluated rates of dementia in ACHD compared with the general population (15). Age at follow up was dichotomized as <65 or >65 years of age. The hazard ratio for all-cause dementia in the ACHD group vs. health controls was 1·61 (95%CI 1·29–2·02). Adults with severe lesions were at greater risk (HR 1·96; 95%CI 1·15–3·34) than those with moderate lesions (HR 1·50; 95%CI 1·14–1·97). Risk was highest for early-onset (<age 65), rather than later-onset dementia (HR early 2·59; 95%CI 1·76–3·81; HR late 1·32; 95%CI 1·00–1·75). This effect persisted after propensity-score matching for acquired cardiovascular disease and diabetes mellitus (15).

The compounding longitudinal effects of cerebrovascular disease on the already susceptible brains of the CHD population are not well characterized, and mechanisms are not fully understood. In addition to the known cognitive burden of clinically evident embolic events that can occur with structural heart disease, there may be additive pathological mechanisms, particularly those with dysrhythmias or congestive heart failure, similar to those described in the atrial fibrillation and heart failure population, including chronic hypoperfusion, subclinical microemboli, and ongoing inflammation outside of the effects of overt ischemic events (5, 18, 94).

## Management

### Stroke Prevention

The literature underlying recommendations for stroke prevention in the ACHD population is primarily observational. Results may be confounded by measured or unmeasured patient characteristics and choice of who is included and excluded. There may be biases, amongst other reasons, by choice of treatment allocations and outcome assessments. Prospective, randomized trials are required to guide therapy based on a higher level of evidence.

#### Antithrombotics

Current guideline recommendations for antithrombotic strategy in ACHD and post-Fontan patients are outlined in Table 4 (22, 50, 95, 96). and Table 5 (10, 50, 95), respectively. Established schema for risk stratification in acquired heart disease and atrial fibrillation/flutter (such as the CHADS_2_ and CHA_2_DS_2_VASc scores) do not apply in CHD as they fail to consider the presence, type, or severity of CHD lesions. In CHD the approach is thus modified to consider the underlying condition. With severe lesions, anticoagulation is consistently recommended in the presence of AF/IART, irrespective of the presence or absence of other risk factors for thromboembolism. In those with moderately complex lesions and AF/IART, anticoagulation is generally recommended. For those with simple CHD and AF/IART then it is recommended that antithrombotic therapy be guided by the aforementioned established stroke risk scores (22, 50, 95, 96). Outside of the Fontan population, only the 2013 AHA Scientific Statement on Prevention and Treatment of Thrombosis in Pediatric and Congenital Heart Disease addresses antithrombotics in the context of secondary stroke prevention (50). In contrast to the non-CHD population, VKA are the preferred anticoagulant agent for patients with moderate or severe CHD owing to insufficient safety and efficacy data with non-Vitamin K antagonist oral anticoagulants (NOACs) in this population (22, 50, 95, 96). However, there is some observational literature reporting experience with NOACs (see following section).

**Table 4:** Consensus recommendations on antithrombotics for prevention of stroke and systemic embolism in ACHD.

**Guideline**	**Recommendation**	**Level of evidence**
European Society of Cardiology–Guideline for the management of grown-up congenital heart disease−2010 (95)	Currently available data do not support any benefit of routine anticoagulation or antiplatelet therapy in cyanotic patients to prevent thromboembolic complications. There is, however, increased risk of bleeding Indications for anticoagulation–A. fib/flutter. Target INR 2.0–2.5.	None provided
AHA Scientific Statement–Prevention and Treatment of Thrombosis in Pediatric and Congenital Heart Disease−2013 (50)	Warfarin is recommended for ACHD with paroxysmal, persistent, or permanent A. fib/flutter or IART, or history of embolic stroke	Class I; Level C
PACES/HRS Expert Consensus Statement on the Recognition and Management of Arrhythmias in Adult Congenital Heart Disease−2014 (96)	Adults with complex CHD and sustained or recurrent IART or AF should receive long-term oral anticoagulation For adults with moderate complexity CHD and sustained or recurrent IART or AF, long-term oral anticoagulation is reasonable	Class I; Level B Class IIa; Level C
AHA Scientific Statement–Congenital Heart Disease in the Older Adult−2015 (22)	Anticoagulation with warfarin is recommended in older (>40) ACHD patients with sustained A.fib whether or not those patients meet usual criteria for a.fib/flutter in acquired heart disease (eg., CHADS_2_ scoring).	Class I; Level C

**Table 5:** Consensus recommendations on antithrombotics for prevention of stroke and systemic embolism in post-Fontan patients.

**Guideline**	**Recommendation**	**Level of evidence**
European Society of Cardiology–Guideline for the management of grown-up congenital heart disease−2010 (95)	In adults with Fontan, anticoagulation is indicated in the presence of atrial thrombus, atrial arrhythmias, or thromboembolic events.	Not provided
AHA Scientific Statement–Prevention and Treatment of Thrombosis in Pediatric and Congenital Heart Disease−2013 (50)	Warfarin is recommended in adult patients with Fontan circulation who have a documented atrial thrombus, atrial arrhythmia, or a thromboembolic episode.	Class I; Level C
	Long-term warfarin therapy is reasonable for primary stroke prevention in adults with Fontan circulation who have a documented atrial-level shunt.	Class IIa; Level C
	Long term antiplatelet therapy for prevention of thrombosis is reasonable after the Fontan procedure.	Class IIa; Level C
	Long term therapy with warfarin may be reasonable post-Fontan for patients with anatomic or hemodynamic risks.	Class IIb; level C
AHA Guideline for the Management of Adults with Congenital Heart Disease−2018 (10)	Anticoagulation with a vitamin K antagonist is recommended for adults with Fontan palliation with known or suspected thrombus, thromboembolic events, or prior atrial arrhythmia.	Class I; Level–consensus
	Antiplatelet therapy or anticoagulation with a vitamin K antagonist may be considered in adults after Fontan palliation without known or suspected thrombus, thromboembolic events, or prior arrhythmia.	Class IIb; Level–moderate non-randomized

In Fontan patients, VKA anticoagulation is recommended for atrial tachyarrhythmias, atrial thrombus, or history of thromboembolism. Recommendations for antithrombotics for primary prevention in Fontan patients beyond these indications are less consistent, with some advocating for empiric anticoagulation in the presence of significant intracardiac right-to-left shunt or veno-veno collaterals. VKA are preferred in the Fontan population owing to concerns regarding the safety of NOACs in the presence of Fontan-associated hepatic impairment and altered baseline coagulation (10, 50, 95, 96).

A small body of observational literature and one prospective randomized trial compares antithrombotic regimens in pediatric post-Fontan patients (97). The literature suggests that post-Fontan patients on antithrombotics have a lower risk of thromboembolic events than those on no treatment. However, whether anticoagulation confers superior protection to antiplatelet therapy is unclear. One study of long-term thromboembolic risk in 261 post-Fontan patients with a median follow-up of 25 years found that after the initial post-operative risk period, there was a plateau, with the risk of thromboembolic death then increasing sharply again at 15 years post-intervention (98). Thrombosis-free survival was 98·7% at 10 years, and 90·8% at 25 years post-Fontan. Absence of antithrombotic use for primary prevention of thrombosis was a powerful independent predictor of mortality, though confidence intervals were wide (HR 91·6; 95%CI 4·2–2004·8).

This same group of patients was also evaluated in a cohort study evaluating different antithrombotic regimens (99). Half were treated with no antithrombotic medication, and the remaining half were split equally between aspirin and VKA therapy. INR data was not routinely collected. There was a significantly higher thromboembolic event rate in the no-treatment group, with no significant difference between the aspirin and VKA groups. In both antithrombotic groups, 20-year freedom from thromboembolic events was 86%, compared to 52% in the no-treatment group (HR 8·49; 95%CI 3·63–19·86).

In a cohort of 278 adult post-Fontan patients (mean age 31) with atrial tachyarrhythmias, 65% were treated with antiplatelets, 33% with anticoagulation, and 2% on no therapy (40). Over 1,500 patient-years, there were significantly fewer thromboembolic events in the anticoagulated group compared to those on antiplatelet or no antithrombotic therapy (HR 0·63; 95%CI0·32–0·79).

One prospective trial randomized 111 pediatric post-Fontan patients to aspirin vs. VKA. Over a 2-year follow-up period, 19% in both groups had a thromboembolic event, with one major bleeding event in the VKA group (97).

#### Use of NOACs in ACHD

The limited body of literature describing the experience with NOACs in ACHD reports a low thromboembolic risk and higher rates of non-major bleeding, though proportions of Fontan patients and duration of follow-up varies.

A retrospective series followed 21 adult Fontan patients (median age 33) treated with NOACs over 316 patient-months (100). Primary indication for anticoagulation was atrial dysrhythmia (11 patients), previous thrombosis (eight), and persistent right-to-left shunt (two). Over the course of the study, one venous thromboembolism and one new atrial thrombus occurred. Ten patients experienced minor bleeding, and one died due to out-of-hospital cardiac arrest.

A recent prospective study followed 75 ACHD patients (median age 51, range 22–74) taking NOACs (101). Indications for anticoagulation were primary prevention of thromboembolism in the context of atrial dysrhythmia (76%), secondary stroke prevention (20%), and the remainder for a history of venous thromboembolism or known atrial thrombus. Complex ACHD lesions were present in 21% of the group and 7% of patients were cyanotic, including 3 Fontan patients. Over a mean of 12 months, two disrupted therapy due to bleeding, 47% had minor bleeding, and there were no thrombotic complications.

A prospective registry study evaluated adverse events in 99 ACHD patients with atrial dysrhythmias in the first month of initiating NOACs (102). Median age was 49 years, 29% had complex lesions, and 11% had Fontan circulations. One-fifth had a previous arterial or venous thromboembolic event and 55% were transitioned from a VKA. At 1 month, there were no thromboembolic events and 8 minor adverse events (including minor bleeding and other side effects).

#### Vascular Risk Factor Management

There are no randomized trials to guide management, nor is there specific evidence on the effects of optimization of acquired vascular risk factors such as hypertension, dyslipidemia, and diabetes in ACHD (103). The American Heart Association (AHA) has published guidelines addressing management goals and vascular risk factor management specific to adults with congenital heart disease (10, 104). as well as guidelines for the management of CHD in the Older Adult (22).

The guidelines make some specific recommendations for management of hypertension. General population guidelines are extrapolated to ACHD, with consideration given to lower blood pressure targets in those with aortic dilatation or lesions benefitting from lower afterload (single ventricle, systemic RV). Recommended pharmacotherapy is as per general population guidelines, with caveats for cyanotic patients (angiotensin-converting enzyme [ACE] inhibitors and diuretics should be limited to necessary instances and monitored closely), Eisenmenger physiology (extreme caution with vasodilating agents that could accentuate right to left shunting), and residual aortic coarctation (ACE inhibitors may precipitate acute renal failure and should be used with vigilance) (22).

The guidelines suggest those >40 years of age or with BMI >25 kg/m^2^ be screened for abnormal glucose tolerance, and reassessed every 3 years if normal. Blood pressure targets for ACHD patients with diabetes and lipid targets follow those for the general population (22).

### Acute Stroke

There are no guideline recommendations regarding acute interventions for ACHD patients with acute stroke. This pertains to thrombolysis and thrombectomy for ischemic stroke, as well as supportive measures such as fluid, positioning, and blood pressure management. The existing pediatric literature is as sparse. Interventional therapies for pediatric CHD patients with acute stroke, including thrombolytics and thrombectomy, have been reported as part of case series, though time to intervention is most often outside of optimal therapeutic windows or is not reported (103, 105, 106).

As with all patients being considered for thrombolysis, it should be clarified if the patient is taking anticoagulants, and if there have been any disruptions to this regimen. There would not typically be arterial anatomic differences that would preclude standard endovascular access and approaches for mechanical thrombectomy, though repeat or recent catheterizations may complicate vascular access. Complications of thrombectomy specific to this population are also not well represented in the literature (107). In general, considerations should be made with regards to patients' individual anatomy, surgical and procedural history, and potential issues related to tenuous hemodynamics and risk for arrhythmias (41, 108–110). Emergent expert cardiology and anesthesia consultation should be sought for periprocedural guidance.

## Directions for Future Research

The ACHD population is growing, and these individuals are at increased risk for cerebrovascular disease. The burden of disease and risk factors for progression are incompletely understood in the absence of large, high-quality longitudinal studies including brain and vascular neuroimaging and cognitive assessment. This information will be essential in developing risk stratification schema that may subsequently guide studies of targeted primary and secondary prevention. Organized efforts to collect experience on acute stroke treatment in these individuals may also be helpful in developing periprocedural pathways for mechanical thrombectomy (107). As with all rarer diseases, large international cooperative efforts should be pursued to best serve these individuals.

Given the increased risks of cerebrovascular disease in ACHD, neurologists have a role in assessment and management in this group. The recently published 2018 AHA guidelines for the management of ACHD do not include neurologist involvement or routine neuroimaging as part of multidisciplinary care and follow-up. The guideline section for cyanotic heart disease mentions performing “cerebral imaging for any new headache or neurologic sign to assess for cerebral abscess, hemorrhage or stroke.” There may be a role for proactive baseline neuroimaging to assess burden of infarcts and markers of cerebral small vessel disease (including burden of white matter hyperintensities and microbleeds) to ascertain risk of recurrent stroke, cognitive impairment and intracranial bleeding (111–115), and may help to guide changes in secondary prevention strategies. Repeat follow-up neuroimaging in high-risk patients to assess for subclinical events may also help to identify patients in need of alternative antithrombotic strategies or closer monitoring. In addition to interpreting neuroimaging in the context of a patient's history, neurologists have a role in performing baseline and follow-up clinical assessments, including screening for new or progressive cognitive concerns that would warrant further workup. Improved collaboration between neurologists and cardiologists may help to optimize the brain health of the ACHD population.

## Conclusion

Congenital heart disease, particularly in post-Fontan and cyanotic individuals, confers a significant risk for stroke and vascular cognitive impairment throughout the lifespan. Characterization of this risk requires careful consideration of a patient's unique, and sometimes dynamic, physiology, in addition to conventional vascular risk factor monitoring and management. Improved awareness of relevant risks is needed amongst adult neurologists. A greater role for neurologists in the routine management of ACHD will help to improve patients' lifespan, brain health and quality of life, both now and in the future (Table 6).

**Table 6:** Take-home considerations.

ACHD patients with complex lesions are at high risk of ischemic and hemorrhagic stroke. ACHD patients have specific anatomic, electrophysiological and systemic risks for cerebrovascular disease that are distinct from the general population. A detailed understanding of an individual patient's anatomy and surgical history may provide important clues regarding the mechanisms of cerebrovascular insults. ACHD patients also are at risk for conventional vascular risk factors, though these risks may interact with their underlying cardiac disease, and management may differ than in conventional patients. Outpatient ACHD patients may benefit from early involvement of subspecialty Cardiologists and Stroke Neurologists familiar with these complex disorders to help in the understanding and optimization of potential risk factors. In a hyperacute stroke presentation, there are no guidelines or substantive evidence to guide thrombolysis, thrombectomy, blood pressure targets, fluid management, and patient positioning. Early steps in the evaluation and acute management of an ACHD patient should include: ◦ Antithrombotic history: even young patients with ACHD may be anticoagulated ◦ Clarifying the anatomy: a detailed description of the cardiac lesion and the surgical history will be integral to managing hemodynamics and determining the feasibility of a catheter approach if thrombectomy is attempted. ◦ Early discussion with cardiologists and anesthetists with ACHD expertise. ◦ Blood pressure targets and agents should be decided upon in consultation with subspecialists. Patients with shunt lesions require cautious changes in blood pressure and team decision-making.

## Author Contributions

JS contributed to literature search and review, manuscript writing, and figures. JA and DH contributed to manuscript and literature review. TSF contributed to supervision of article design and process. All authors contributed to literature search and review, as well as manuscript writing.

### Conflict of Interest Statement

JA reports grants and personal fees from Bayer, grants and personal fees from BMS/Pfizer, grants and personal fees from Servier outside the submitted work. TSF: Bayer Canada (study medication for an unrelated indication). The remaining authors declare that the research was conducted in the absence of any commercial or financial relationships that could be construed as a potential conflict of interest.
